# Leukemia inhibitory factor receptor is a novel immunomarker in distinction of well-differentiated HCC from dysplastic nodules

**DOI:** 10.18632/oncotarget.3136

**Published:** 2015-02-05

**Authors:** Qin Luo, Yurong Zhang, Ning Wang, Guangzhi Jin, Haojie Jin, Dishui Gu, Xuemei Tao, Xisong Huo, Tianxiang Ge, Wenming Cong, Cun Wang, Wenxin Qin

**Affiliations:** ^1^ State Key Laboratory of Oncogenes and Related Genes, Shanghai Cancer Institute, Renji Hospital, Shanghai Jiao Tong University School of Medicine, Shanghai, China; ^2^ Department of Pathology, Eastern Hepatobiliary Surgery Hospital, Second Military Medical University, Shanghai, China

**Keywords:** hepatocellular carcinoma, leukemia inhibitory factor receptor, high-grade dysplastic nodules, well differentiated-small hepatocellular carcinoma

## Abstract

Differential diagnosis of well-differentiated hepatocellular carcinoma (WD-HCC) and high-grade dysplastic nodules (HGDNs) represents a challenge for pathologists. Several immunohistochemistry markers have been identified to distinguish hepatocellular carcinoma (HCC) from HGDNs. However, sensitivity or specificity of the individual marker is still limited. In this study, we analyzed dynamic alteration of leukemia inhibitory factor receptor (LIFR) and CD34 during hepatocarcinogenesis from dysplastic nodules to small HCC. The diagnostic performance of LIFR and CD34 combination in WD-HCC and HGDNs was investigated by logistic regression models and validated in an independent validation cohort. LIFR was decreased and CD34 was increased along with stepwise progression of hepatocarcinogenesis from low-grade dysplastic nodules (LGDNs) to small HCC. The sensitivity and specificity of the LIFR and CD34 combination for WD-HCC detection were 93.5% and 90.5%, respectively. In addition, colony formation assay was used to explore the role of LIFR in tumorigenesis. Silencing of LIFR could significantly promote colony formation of HCC cells, whereas ectopic overexpression of LIFR resulted in impaired ability of colony formation of HCC cells. These findings indicate that LIFR and CD34 combination may be used as an available differential diagnostic model for WD-HCC from HGDNs in clinical practice.

## INTRODUCTION

Hepatocellular carcinoma (HCC) is the fifth most frequent cancer and the third leading cause of cancer-related death worldwide [1, 2]. More than 90% of HCC patients have a cirrhotic background because of hepatitis B or C infection, metabolic syndrome, or alcohol abuse [3]. HCC classically develops on cirrhosis through a multistep process of carcinogenesis. Recently, the multistep process of hepatocarcinogenesis is ranged from benign regenerative nodules (RNs) to low-grade dysplastic nodules (LGDNs) and high-grade dysplastic nodules (HGDNs), and eventually to early HCC (eHCC) and advanced HCC [4].

Among precancerous lesions developed on cirrhosis, the risk of malignant transformation has been significantly increased at approximately 20% for LGDNs and 20%–80% for HGDNs [5, 6]. However, differential diagnosis among these lesions based on histological criteria alone is very difficult even for experienced pathologists, especially the liver biopsies or surgical specimens involving HGDNs and well differentiated-sHCC (WD-sHCC) [7, 8]. Several immunohistochemistry markers, including heat shock protein 70 (HSP70), glypican 3 (GPC3), glutamine synthase (GS), CD31, and CD34, have been identified to distinguish HCC from HDGNs [7–10]. However, sensitivity or specificity of the individual marker is still limited. For instance, the sensitivity for distinguishing WD-sHCC from HGDNs was only 78.1% for HSP70, 59.4% for GS and 68.8% for GPC3, respectively [8]. In addition, CD34 positive capillaries could also occur in HGDNs, which may influence the accuracy of pathological diagnosis [10]. Therefore, it is urgent to identify novel and reliable immunohistochemistry markers for differential diagnosis between HGDNs and WD-sHCC.

Leukemia inhibitory factor receptor (LIFR) is an integral component of glycoprotein130-LIFR complex and participates in signal transduction through interleukin-6 (IL-6) cytokine family [11]. The biological roles of interleukin-6 (IL-6) cytokine family are widely different, ranging from maintenance of stem cell pluripotency, glucose uptake, hepatoprotective activities, to modulation of cell proliferation. Chen *et al* identified LIFR as a metastasis suppressor which exerted its function through Hippo-YAP pathway [12]. In addition, expression of LIFR inversely correlated with metastasis and clinical outcomes of breast cancer patients. Iorns *et al* also identified LIFR as a novel suppressor of breast tumor through whole genome *in vivo* RNAi [13]. Furthermore, it has been reported that LIFR is a tumor suppressor gene in HCC and its down-regulation in tumor tissues is mostly dependent on promoter hypermethylation [14, 15]. According to the oncomine data-mining analysis, down-regulation of LIFR expression was found in several types of cancer, including breast cancer, colorectal cancer, gastric cancer, liver cancer, *etc*. More attractively, expression of LIFR was significantly decreased from liver cell dysplasia to hepatocellular carcinoma in Wurmbach's dataset [16]. However, whether LIFR could be an immunomarker in distinction of WD-sHCC from HGDNs has not been investigated.

In the present study, we analyzed the expression patterns of LIFR and CD34 in liver cirrhosis (LC), LGDNs, HGDNs, WD-sHCC, and moderately differentiated HCC (MD-sHCC). The diagnostic accuracy of LIFR combined with CD34 was further evaluated. Moreover, diagnostic model for distinguishing WD-sHCC from HGDNs was established by logistic regression analyses and validated in an independent validation cohort. Our results suggest that LIFR and CD34 combination is a reliable diagnostic model in distinction of WD-sHCC from HGDNs.

## RESULTS

### Identification of LIFR as a potential biomarker through oncomine data-mining analysis

Down-regulation of LIFR was found in 12 of 20 cancer types, especially cancers of digestive system, such as liver cancer, colorectal cancer, and gastric cancer, through oncomine data-mining analysis (Figure 2A). In Wurmbach's dataset, we found that level of LIFR mRNA was significantly decreased from liver cell dysplasia to hepatocellular carcinoma with all four probes (Figure 2B).

**Figure 1:**
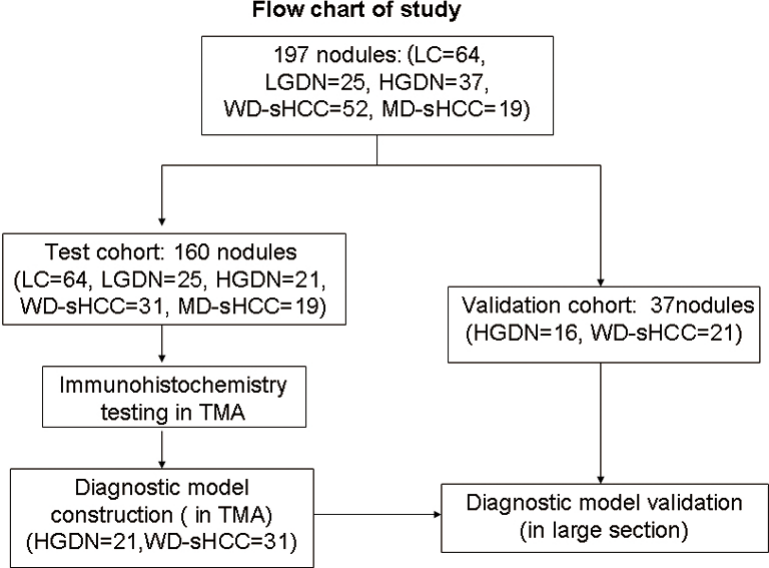
Flow chart of nodule enrollment In the test cohort, 160 nodules (LC = 64, LGDN = 25, HGDN = 21, WD-sHCC = 31, and MD-sHCC = 19) were used in expression profiles study. Diagnostic models were constructed using 52 nodules (HGDN = 21, WD-sHCC = 31) from the expression profiles study. Diagnostic model validation used an independent set of large section, which contained 16 HGDNs and 21 nodules of WD-sHCC.

**Figure 2:**
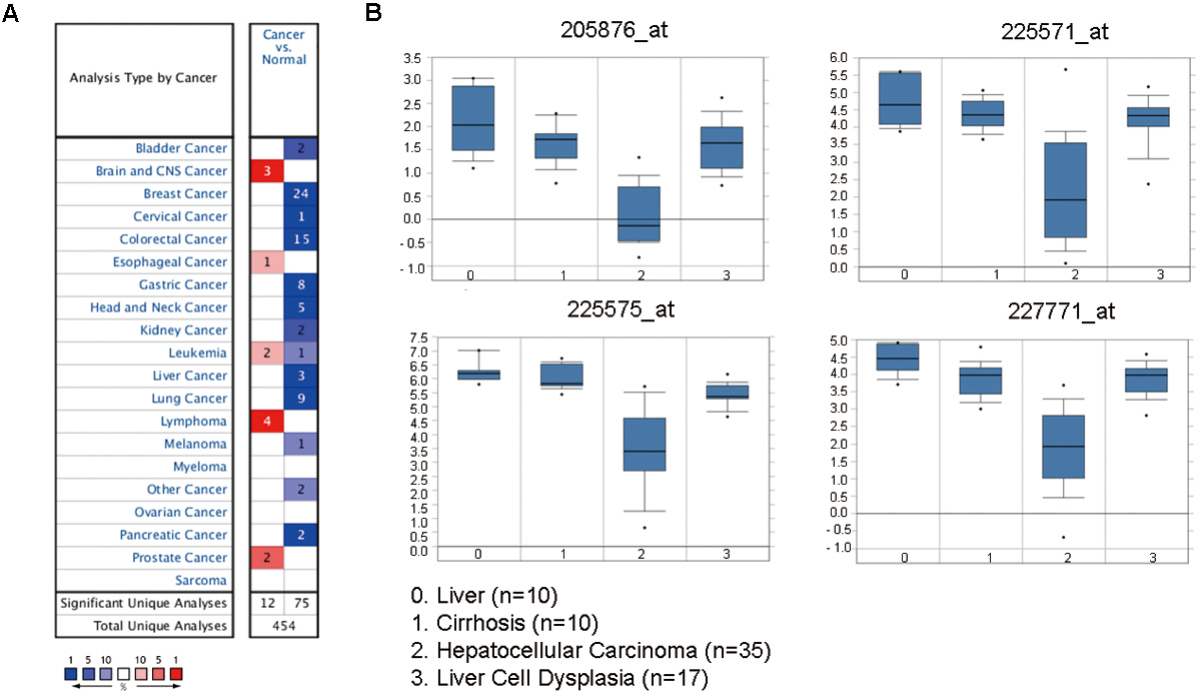
The LIFR mRNA is down-regulated in liver cancer compared with liver cell dysplasia as revealed by oncomine data-mining analysis **(A)** Down-regulation of LIFR was found in 12 of 20 cancer types. **(B)** Level of LIFR mRNA was significantly decreased from liver cell dysplasia to HCC in all of the four probes (205876_at, 225571_at, 225575_at, 227771_at) in Wurmbach's dataset.

### Down-regulation of LIFR during malignant progression of HCC

**Figure 3:**
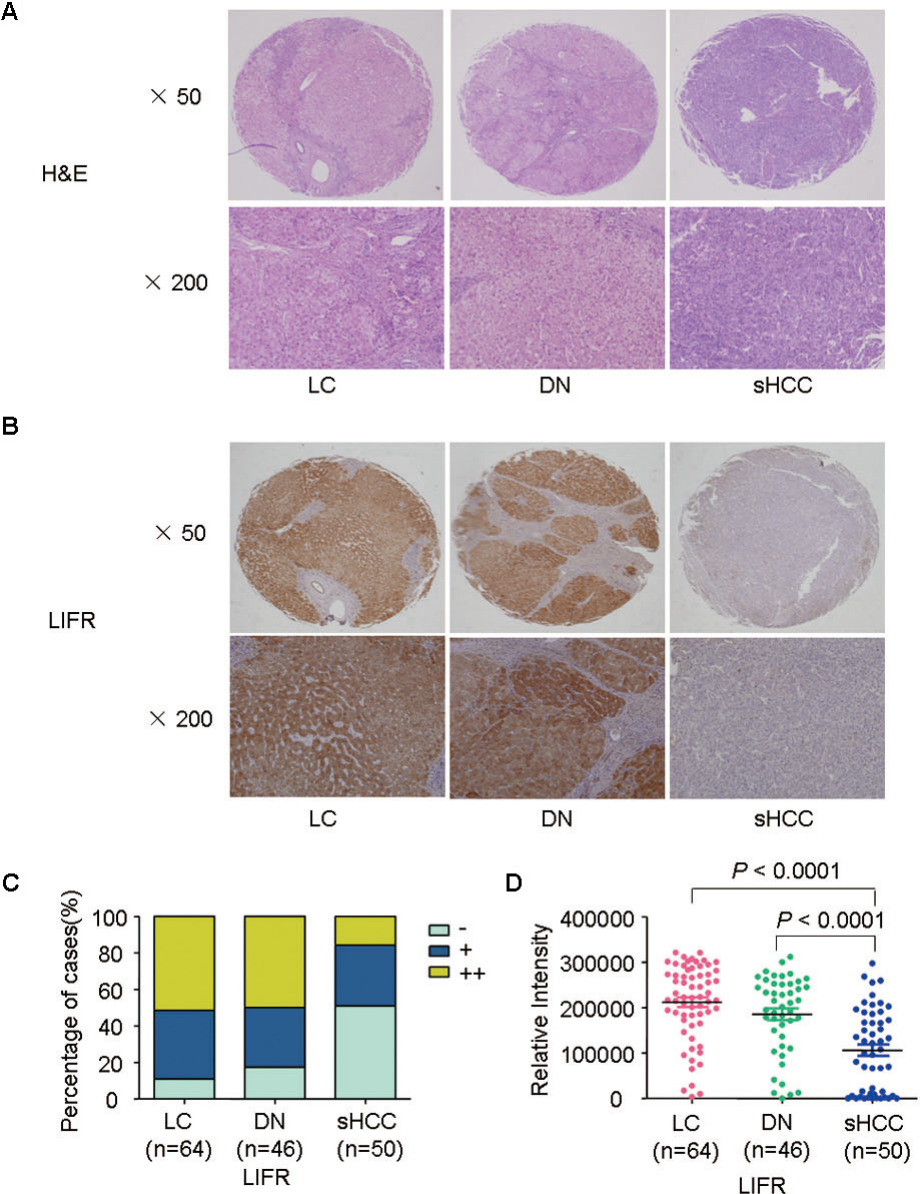
Representative images of HE staining and LIFR expression for LC, DN, and sHCC **(A)** Typical HE-stained images for LC (*n* = 64), DN (*n* = 46), and sHCC (*n* = 50). **(B)** Immunostaining of LIFR for LC, DN, and sHCC. **(C)** Immunostaining scores distribution of LIFR expression in LC, DN, and sHCC. **(D)** A scatter plot of IOD for LIFR was obtained from tissue microarray.

In addition to oncomine data, we observed that level of LIFR in sHCC were lower than that in LC and DN through immunohistochemistry staining. Representative images of HE staining and LIFR expression were shown in Figure 3A–3B. The immunoreactivity score distribution of LIFR significantly decreased during hepatocarcinogenesis from DN to sHCC (Figure 3C). The negative immunoreactivity was demonstrated in 10.94% of LC, 17.39% of DN and 52% of sHCC (Figure 3C). Based on analysis of IOD, we found that level of LIFR was significant lower in sHCC than that in LC and DN (Figure 3D, *P* < 0.0001).

**Figure 4:**
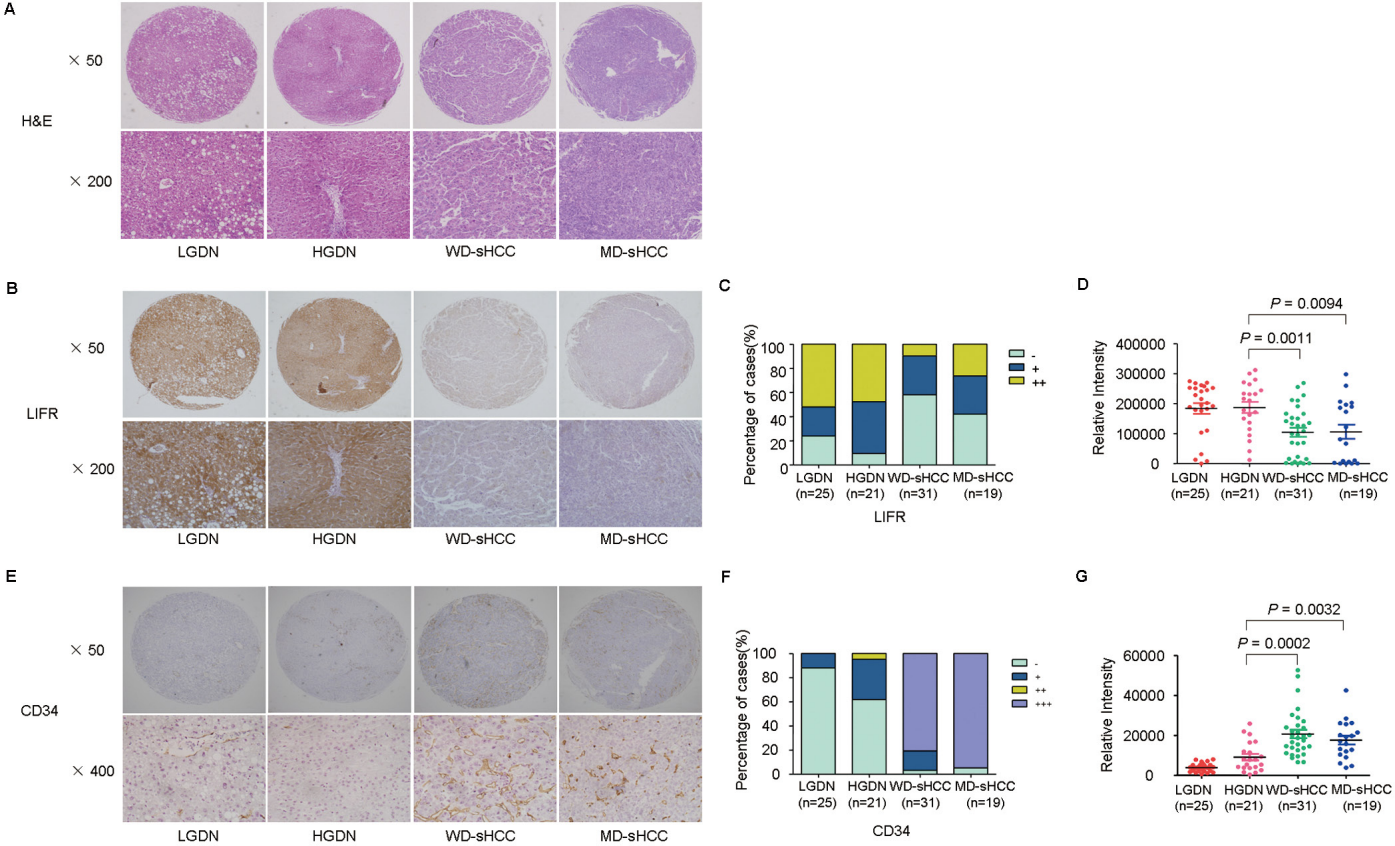
Representative images of HE staining and immunohistochemical staining of LIFR and CD34 in LGDN, HGDN, WD-sHCC, and MD-sHCC **(A)** Typical HE-stained images for LGDN (*n* = 25), HGDN (*n* = 21), WD-sHCC (*n* = 31), and MD-sHCC (*n* = 19). **(B)** Immunostaining of LIFR in LGDN, HGDN, WD-sHCC, and MD-sHCC. **(C)** Immunostaining scores distribution of LIFR expression. **(D)** Immunohistochemical expression of LIFR in LGDN, HGDN, WD-sHCC, and MD-sHCC. A scatter plot of IOD for LIFR was obtained from tissue microarray. **(E)** Immunostaining of CD34 in LGDN, HGDN, WD-sHCC, and MD-sHCC. **(F)** Immunoreaction score distribution of CD34. **(G)** A scatter plot of IOD for CD34 was obtained from tissue microarray.

Because differential diagnosis between HGDN and WD-sHCC based on morphologic characteristics alone is very difficult for pathologists, we further classified our specimens into LGDN (*n* = 25), HGDN (*n* = 21), WD-sHCC (*n* = 31), and MD-sHCC (*n* = 19). Representative images of HE and LIFR staining were shown in Figure 4A–4B. The negative immunoreactivity was demonstrated in 24% of LGDN, 9.52% of HGDN, 58.06% of WD-sHCC and 42.11% of MD-sHCC (Figure 4C). Decreased level of LIFR in WD-sHCC compared with HGDN was also confirmed based on analysis of IOD (Figure 4D, *P* = 0.0011).

CD34 had a well sensitivity in the differential diagnosis between HGDNs and WD-sHCC [17]. In order to improve the sensitivity of LIFR in detection of sHCC, CD34 was selected as a combined biomarker in our study. We found that positive staining of CD34 was significant increased along with stepwise progression of hepatocarcinogenesis from LGDN to sHCC (Figure 4E–4G).

### Establishment of diagnostic model in test cohort

**Figure 5:**
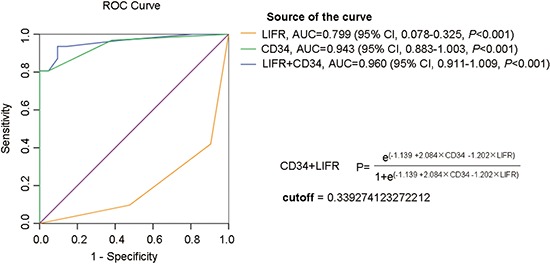
ROC curve analysis of individual marker or combinations of LIFR and CD34 for distinguishing WD-sHCC from HGDN lesions AUC was 0.799 for LIFR, 0.943 for CD34, 0.960 for LIFR and CD34 combinations.

To enhance efficiency of diagnosis, logistic regression analyses were used to construct diagnostic models using immunohistochemistry data of the test cohort: HGDN (*n* = 21) and WD-sHCC (*n* = 31). The cutoff value was determined by ROC curves. As shown in Figure 5, the area under the curve (AUC) was 0.799 for LIFR, 0.943 for CD34. Furthermore, AUC was 0.960 for LIFR + CD34 combination (cutoff value = 0.3393), suggesting that the AUC for the combination was better than that for any individual marker. The diagnostic model was described in Figure 5.

The sensitivity, specificity, and positive and negative predictive values of individual markers, as well as the combined diagnostic model for HGDN and WD-sHCC detection were described in Table 2. High sensitivity (96.8%) and low specificity (61.9%) for diagnosis of WD-sHCC was observed for CD34 alone. The sensitivity and specificity of LIFR (negative) for detection of WD-sHCC were 58.1% and 90.5%, respectively. Sensitivity and specificity for differentiating WD-sHCC and HGDN were 93.5% and 90.5% for LIFR + CD34 combination. Notably, the specificity of CD34 for discriminating between HGDN and WD-sHCC was significantly improved after the combination of LIFR.

**Table 1:** Clinico-pathological features of the patients

Variable	LC	DN	HCC
Patients	64	59	71
No. of nodules	64	62	71
Sex			
Male	47	49	60
Female	17	10	11
Age			
Mean	45.6	53.3	53.4
SD	10.7	9.6	10.1
HBsAg			
Positive	48	54	60
Negative	9	4	9
LC			
Yes	64	48	52
No	-	11	13
Serum AFP			
Positive (≤ 20 ng/ml)	38	33	46
Negative(> 20 ng/ml)	9	25	24
DN grade			
LGDN	-	25	-
HGDN	-	37	-
DN with HCC	-	43	-
Child-pugh class			
A	-	51	64
B	-	5	1
C	-	1	-
TNM			
I	-	-	64
II	-	-	1
III–IV	-	-	-
Tumor differentiation			
Well	-	-	52
Morderate	-	-	19
Poor	-	-	-

LC, liver cirrhosis; LGDN, low grade dysplastic nodule; HGDN, high grade dysplastic nodule; AFP, α-fetoprotein; HBsAg, hepatitis B virus surface antigen; SD, standard deviation; TNM, UICC TNM classification (6th edition).

**Table 2:** Sensitivity, specificity, positive and negative predictive values for WD-sHCC detection using individual markers and marker combinations

	WD-sHCC (*n* = 31)	HGDN (*n* = 21)	Sensitivity	Specificity	PPV	NPV
LIFR negative	18	2	58.10%	90.50%	59.40%	90.00%
CD34 positive	30	8	96.80%	61.90%	78.90%	92.90%
Predicted by LIFR + CD34	29	19	93.50%	90.50%	93.50%	90.50%

WD-sHCC, well differentiated hepatocellular carcinoma; HGDN, high grade dysplastic nodule; PPV, positive predictive value; NPV, negative predictive value.

### Evaluation of diagnostic model in validation cohort

**Figure 6:**
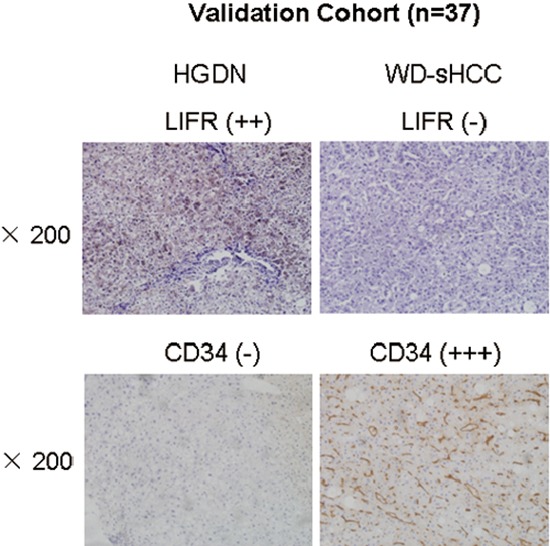
Expression of LIFR and CD34 in validation cohort Representative images of LIFR and CD34 staining in HGDN and WD-sHCC from independent validation cohort (HGDN = 16 and WD-sHCC = 21).

The diagnostic model was used to an independent validation cohort: HGDN (*n* = 16) and WD-sHCC (*n* = 21). Representative immunostaining images of serial large sections for HGDNs and WD-sHCC were shown in Figure 6. Similar to TMA analysis, LIFR was significantly down-regulated in WD-HCC compared with HGDN. CD34 was dramatically up-regulated in WD-HCC in comparison with HGDN. The immunostaining scores for LIFR and CD34 in individual cases were used as an index for the diagnostic model. An output value ≤ 0.3393 is considered highly predictive of HGDNs, while an output > 0.3393 predicts WD-sHCC. Finally, 100% of WD-sHCCs (21/21) and 81.3% of HGDNs (13/16) were correctly diagnosed by LIFR + CD34 combination (Supplementary Table 1).

### LIFR negatively regulates the ability of colony formation of HCC cells

**Figure 7:**
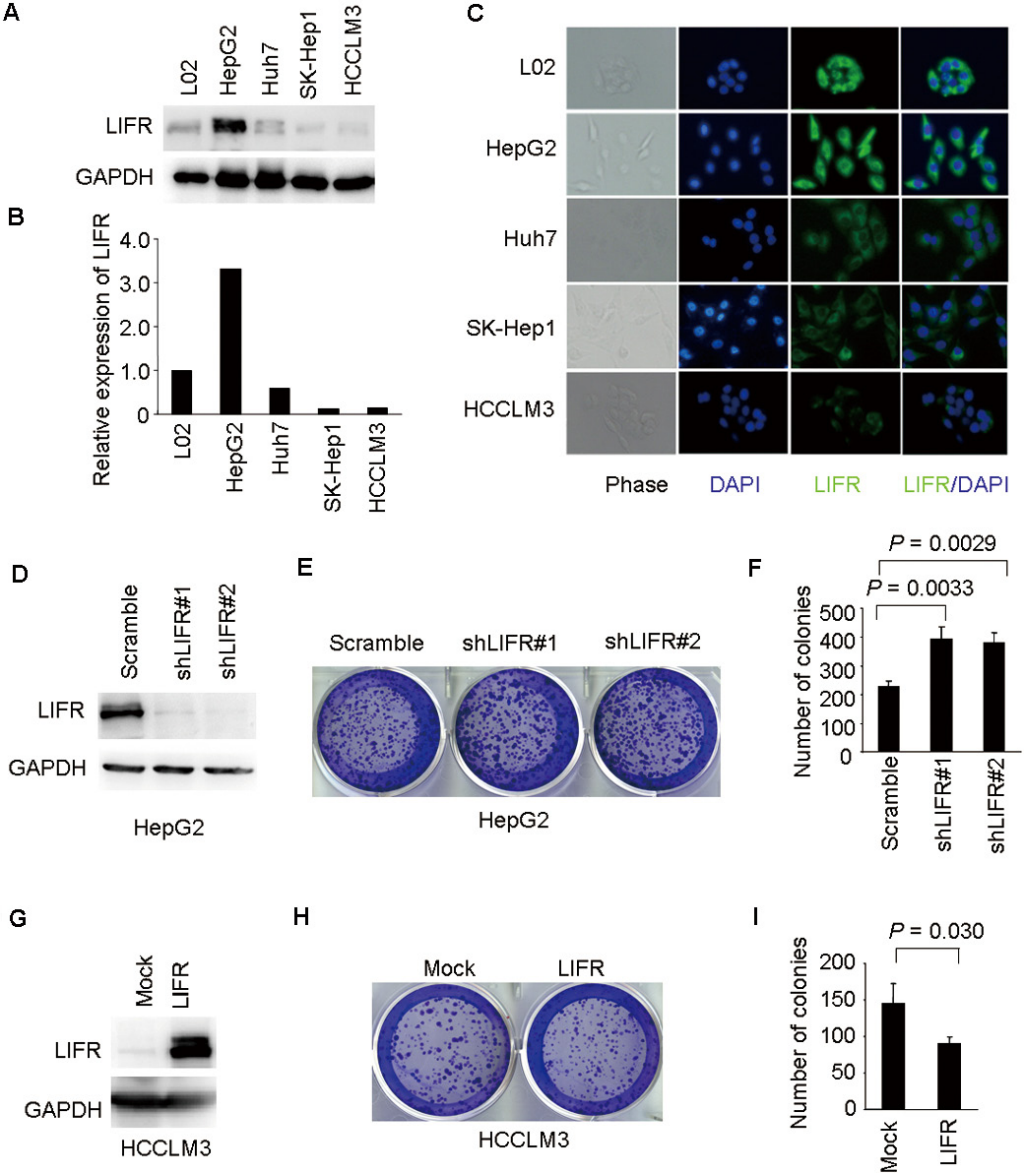
LIFR inhibits ability of colony formation of HCC cells **(A–C)** LIFR expression was measured by western blotting and immunofluorescence analysis in a panel of liver cell lines. **(D)** Western blotting was used to validate knockdown of LIFR in HepG2-shLIFR cells. **(E–F)** knockdown of LIFR facilitated ability of colony formation in HepG2 cells. **(G)** Effective overexpression of LIFR in HCCLM3-LIFR cells was confirmed by western blotting. **(H–I)** Overexpression of LIFR impaired ability of colony formation in HCCLM3 cells.

We examined LIFR expression in a series of HCC cell lines (L02, HepG2, Huh7, SK-Hep1, and HCCLM3). The protein levels of LIFR in L02 and HepG2 cells were higher compared with Huh7, SK-Hep1, and HCCLM3 cells (Figure 7A–7B). Next, similar results were obtained through immunofluorescence staining (Figure 7C).

In order to explore the roles of LIFR in colony formation of HCC cells, we employed lentivirus-mediated shRNA to knockdown LIFR in HepG2 cells and generated a HCCLM3 cell line ectopically overexpressing LIFR. Stable shRNA-mediated knockdown of LIFR in HepG2 cells and effective overexpression of LIFR in HCCLM3 cells were confirmed by western blotting (Figure 7D and 7G). Colony formation assays indicated that silencing endogenous LIFR in HepG2 cells significantly increased the ability of colony formation compared with mock cells (Figure 7E–7F). Similar results were obtained using two different shRNAs targeting different regions of the LIFR mRNA. On the contrary, overexpression of LIFR in HCCLM3 cells dramatically impaired the ability of colony formation (Figure 7H–7I). Collectively, these results indicate that LIFR may play a functional role in hepatocarcinogenesis.

## DISCUSSION

Accumulating evidence strongly indicates the existence of a sequence of events in hepatic nodules during hepatocarcinogenesis, and these nodules are recognized as precancerous lesions of HCC [18, 19]. International Working Party (IWP) classified these nodular lesions into RNs, LGDNs, HGDNs, and HCC [20]. It is not difficult to differentiate LGDNs or other cirrhotic nodules from WD-sHCC. However, if the liver biopsies or surgical specimens involve HGDNs and WD-sHCC, the diagnosis of these lesions is very difficult, even for expert pathologists. These small lesions only have subtle differences from the surrounding parenchyma, which makes them difficult to be diagnosed reproducibly. Several immunohistochemistry markers such as GPC3, HSP70, and GS have been reported in diagnosis of HCC, whereas some limitations still exist. For instance, the sensitivity and specificity of GPC3 for the differential diagnosis were 61.4% and 92%, respectively [7]. Nault *et al* indicated that only 39% of eHCC had two or three positive immunohistochemistry markers (GPC3, HSP70, and GS) [21]. CD34 had a high sensitivity for distinguishing WD-sHCC from HGDNs, however its positive staining may also occur in some confusing cases of HGDNs [10]. In the last decade, several immunohistochemistry markers such as clathrin heavy chain, EZH2, SUOX, ACY1, SQSTM1 and AKR1B10 were used single or in panel to distinguish WD-sHCC from HGDN [17, 22–24]. Somatic TERT promoter mutation was also identified as a new biomarker for transformation of premalignant lesions into HCC [21]. However, sensitivity and specificity of these potential markers still need to be validated.

In the present study, we analyzed the potential of LIFR as an immunohistochemistry maker in distinction of WD-HCC from HGDNs. We used LIFR and CD34 to establish diagnostic panels to differentiate HGDNs from WD-sHCC by using logistic regression analysis, and the models were further tested in an independent validation set of WD-sHCC and HGDNs. The results showed that LIFR and CD34 expression was significantly different between WD-sHCC and HGDNs. Moreover, with combination of LIFR, the specificity of CD34 was significantly improved. The sensitivity and specificity of LIFR + CD34 combination were 93.5% and 90.5%, which were better than that in previous reports, such as the combination of GPC3, HSP70, and GS (Table 2). These findings suggest that CD34 in combination with LIFR could improve the diagnostic accuracy for WD-sHCC detection and may be effective in clinical practice.

LIFR was originally isolated by expression screening of a cDNA library using radioiodinated LIF as a probe [25]. LIFR is structurally related to the IL-6 signal transducer and belongs to the gp130 receptor family. It plays broad roles in cell proliferation, cell differentiation, and maintenance of stem cell pluripotency, *etc*. In Wurmbach's dataset, we found that expression of LIFR was significantly decreased from liver cell dysplasia to hepatocellular carcinoma. Down-regulation of LIFR expression was also observed in several types of cancer and it was identified as tumor suppressor in both HCC and breast cancer [12, 15]. Based on depletion or overexpression experiments, we further found that silencing of LIFR significantly promoted colony formation of HCC cells. In contrast, overexpression of LIFR decreased the ability of colony formation in HCCLM3 cells (Figure 7). Taking into account the importance of colony formation in the tumorigenesis, these results suggested that LIFR may be a functional protein in the malignant transformation of HCC. The detailed mechanism underlying the tumor suppressive effects of LIFR requires further investigation.

In conclusion, we show that LIFR may play a functional role during hepatocarcinogenesis. It is the first time to construct a molecular model by logistic regression for distinguishing WD-sHCC from HGDNs using LIFR and CD34. LIFR and CD34 combination could be used as a differential diagnostic model for WD-sHCC from HGDNs in clinical practice.

## MATERIALS AND METHODS

### Clinical samples and immunohistochemistry staining

197 formalin-fixed paraffin-embedded (FFPE) tissues of liver nodules (LC = 64, DN = 62, and sHCC = 71) were collected retrospectively from patients who underwent curative resection between 2005 and 2011 at the Eastern Hepatobiliary Surgery Hospital (EHBH), Second Military Medical University, Shanghai, China (Table 1). As shown in Figure 1, the test cohort contains 160 specimens: LC (*n* = 64), LGDN (*n* = 25), HGDN (*n* = 21), WD-sHCC (*n* = 31) and MD-sHCC (*n* = 19). Immunostaining scores of HGDN (*n* = 21) and WD-sHCC (*n* = 31) were used in diagnostic model construction. The diagnostic model was used to classify HGDN and WD-sHCC cases in the independent validation cohort: HGDN (*n* = 16), WD-sHCC (*n* = 21). This study was conducted with the approval of Ethics Committee of Renji Hospital, Shanghai Jiao Tong University School of Medicine and Eastern Hepatobiliary Hospital of the Second Military Medical University. Written informed consent from each patient was obtained.

The FFPE tissues were stained with hematoxylin and eosin (HE) and then reviewed by two experienced hepatopathologists (WM Cong and H Dong). LC, LGDNs, and HGDNs were diagnosed according to the criteria described previously. The hepatocytes in LGDNs showed normal or appeared minimal nuclear atypia and slightly increased nucleus to cytoplasm (N:C) ratio, but mitotic figures were absent. HGDNs were defined as having architectural and/or cytologic atypia, but the atypia was insufficient for a diagnosis of HCC. The cytological atypia may have been diffuse or focal and was characterized by nuclear hyperchromasia, nuclear contour irregularities, cytoplasmic basophilia or clear cell change, high N:C ratio, and occasional mitotic figures. Architecturally, the cell plates were thickened by up to three cells, with occasional foci of pseudoglandular formation. All WD-sHCC and MD-sHCC in the diagnostic group were < 3 cm in diameter. WD-sHCC (early HCC) was mainly diagnosed based on the following major histological features proposed by the World Health Organization: (i) increased cell density, more than 2 times that of the surrounding liver, with increased N:C ratio; (ii) irregular, thin trabecular pattern or growth; (iii) pseudoglandular structures; (iv) diffuse fatty change; (v) varying numbers of unpaired arteries; (vi) intratumoral portal tracts; and (vii) stromal invasion.

### Tissue microarrays and immunohistochemistry staining

Tissue microarrays were constructed from 160 specimens: LC (*n* = 64); LGDN (*n* = 25), HGDN (*n* = 21), WD-sHCC (*n* = 31) and MD-sHCC (*n* = 19). Immunohistochemistry staining and integrated optical density (IOD) analysis were performed as previously described [17]. The tissue microarray was stained for expression analysis of LIFR and CD34. Negative and positive controls were included in every immunohistochemistry staining experiments.

Immunostaining scores were independently evaluated by two pathologists who were blinded to the clinical outcome. Immunostaining scoring was performed according to the percentage of positive cells and the intensity of the staining under low magnification (×100). The 3 most representative fields were selected. For LIFR, the immunostaining was scored according to the percentage of positive cells: 0 (0–10%), 1 (11–50%), and 2 (> 51%) and the intensity of the staining: 0 (no staining), 1 (light brown), 2 (brown), and 3 (dark brown). The overall scores were determined using the following formula: overall scores = percentage score × intensity score. Overall scores of ≤ 1, 1–3, and ≥ 3 were defined as −, +, and ++, respectively. For CD34, specimens showing none staining or only a few sinusoids were classified as negative (−), and those showing diffuse staining of sinusoidal endothelium throughout the lesion area were defined as positive. And based on the intensity of immunostaining, they were scored as weak (+), moderate (++), and strong (+++), respectively. Results from the two pathologists showed a high level of consistency for the scoring (more than 90%).

Thereafter, the respective areas were measured at × 200 magnification. IOD of all each photograph was measured using IPP (Image pro-plus) 6.0 software. Data were presented as the mean ± SEM.

### Diagnostic model construction and validation of diagnostic efficiency

Diagnostic model construction and validation of diagnostic efficiency were performed as previously described [17, 22]. Briefly, immunostaining scores of LIFR and CD34 from test cohort (HGDN = 21, WD-sHCC = 31) were used to construct diagnostic model. The scores for LIFR and CD34 were subjected to binary logistic regression to generate diagnostic model for detection of WD-sHCC. The output was the diagnostic score in the range of 0 to 1. During model construction, the diagnostic score of an HGDN lesion was defined as 0, whereas that of a WD-sHCC lesion was defined as 1. The predictive probability of this model was applied to the same data set (HGDN = 21, WD-sHCC = 31). Receiver operating characteristic (ROC) analysis was performed to determine the best cutoff for regression analyses.

The diagnostic model were used to classify HGDNs and WD-sHCC in the validation cohort (HGDN = 16, WD-sHCC = 21). The immunostaining scores of LIFR and CD34 of individual cases were subjected to the diagnostic model. The diagnostic scores were used as an index for classifying WD-sHCC and HGDN.

### Cell culture

Human normal liver cell line L02 was obtained from Shanghai Institute of Cell Biology, Chinese Academy of Sciences. The human HCC cell line HepG2 and SK-Hep1 were purchased from the American Type Culture Collection (ATCC, VA, USA). HCC cell line HCCLM3 was established by Liver Cancer Institute of Fudan University. All the cell lines were cultured in Dulbecco's Modified Eagle's Medium (DMEM) supplemented with 10% fetal bovine serum (FBS) and antibiotics (100 U/ml penicillin, 100 mg/ml streptomycin), in a 5% CO_2_ atmosphere at 37°C.

### Western blotting and immunofluorescence

Protein extraction and western blotting were performed as previously described. Briefly, cell lysates were extracted with T-PER tissue protein extraction reagent (Pierce, Rockford, IL). Equal amounts of total proteins (20 μg) were separated by 10% SDS-PAGE and transferred onto PVDF membrane using a Bio-Rad SemiDry apparatus. After blocking for nonspecific binding, the membranes were incubated with anti-LIFR (1:200 dilution; Santa Cruz Biotechnology) or GAPDH (1:5000 dilution; Kang-Chen, Shanghai, China) overnight at 4°C, followed by HRP-conjugated secondary antibodies for 1 h at room temperature. After washing three times in TBST, protein bands were visualized using chemiluminescence detection.

For immunofluorescence staining, cells were grown on glass coverslips. After an attachment period of 24 h, cells were fixed in 4% paraformaldehyde for 30 min and permeabilized using 0.5% Triton X-100 for 10 min. After blocking with 10% donkey serum in PBS for 1 h, cells were incubated with the primary antibody anti-LIFR (1:100 dilution; Santa Cruz Biotechnology) overnight at 4°C. After thorough washing, cells were then incubated with Alexa-Fluor 488 donkey anti-rabbit IgG (1:100 dilution, Life Technologies) for 30 min. Finally, cells were washed and stained with DAPI. Images were captured using a Leica fluorescence microscope.

### Lentiviral shRNA knockdown and overexpression experiments

To generate cell lines stably overexpressing LIFR, based on the LIFR sequence (NM_001127671.1), the cDNA of LIFR ORF sequence was obtained by linking two PCR amplified products (a and b). The cloning primers are as follows: (a) forward: 5′-CGGATCCATGATGGATATTTACGTATG-3′, and reverse: 5′-TTGTGACATTCCGCTGCTCTTG-3′; (b) forward: 5′-CAAGAGCAGCGGAATGTCACAA-3′, and reverse: 5′- CGGAATTCTTAATCGTTTGGTTTGTTC-3′. Those two cDNA products were mixed with equal molar ratio, and amplified using the forward primer of “a” and the reverse primer of “b”. The cDNA of LIFR ORF sequence was obtained and cloned into the lentiviral expression vector pWPXL (Addgene). The human LIFR shRNA clones were purchased (Thermo Fisher), and the clone numbers are as follows: V3LHS_347496 (designated as ‘shLIFR#1′) and V3LHS_347498 (designated as ‘shLIFR#2′). The pGIPZ-GFP lentiviral vector with a scrambled sequence which does not target any mRNA was used as a negative control.

Viral packaging was performed by co-transfection of pWPXL, pWPXL-LIFR, shLIFR#1, or shLIFR#2 with the packaging plasmid psPAX2 and the envelope plasmid pMD2.G (Addgene) using Lipofectamine 2000 (Invitrogen) in HEK 293T cells. Viruses were harvested at 48 h after transfection, and viral titers were determined. HepG2 and HCCLM3 were infected with 1 × 10^6^ recombinant lentivirus-transducing units in the presence of 6 μg/ml polybrene (Sigma).

### Colony formation assay

For colony formation assays, 1000 cells (HepG2-shLIFR, HCCLM3-LIFR and their relative mock cells) were plated onto 6-well plates and incubated at 37°C for about 2 weeks. When the cells grew to visible colonies, the cells were washed twice with PBS and fixed in 4% paraformaldehyde for 30 min. Then, cells were stained with crystal violet, and the numbers of colonies per well were counted.

### Statistical analysis

Statistical analysis was performed with SPSS 15.0 for Windows (SPSS, Chicago, IL). Quantitative variables were analyzed by Student *t* tests. ROC curves were used to determine the diagnostic values of the markers. *P* < 0.05 was considered statistically significant.

## SUPPLEMENTARY TABLE


