# Supplementary material for: Leukemia inhibitory factor receptor is a novel immunomarker in distinction of well-differentiated HCC from dysplastic nodules
Source: Oncotarget. 2015 Feb 5;6(9):6989–99. doi: 10.18632/oncotarget.3136 (PMC4466664; doi:10.18632/oncotarget.3136)
Supplement: Supplementary file 1 [file oncotarget-06-6989-s001.pdf]

SUPPLEMENTARY TABLE

Supplementary Table 1: Histological diagnosis and diagnostic model diagnoses of the 37 nodules

No	Histology	IHC score		Predict by	
		LIFR	CD34	Vaule	LIFR + CD34
1	HGDN	2	3	0.9375	WDHCC
2	HGDN	2	0	0.0281	HGDN
3	HGDN	1	0	0.0878	HGDN
4	HGDN	2	0	0.0281	HGDN
5	HGDN	1	3	0.9804	WDHCC
6	HGDN	0	0	0.2425	HGDN
7	HGDN	2	2	0.6513	WDHCC
8	HGDN	2	0	0.0281	HGDN
9	HGDN	2	1	0.1886	HGDN
10	HGDN	2	0	0.0281	HGDN
11	HGDN	2	0	0.0281	HGDN
12	HGDN	1	0	0.0878	HGDN
13	HGDN	2	0	0.0281	HGDN
14	HGDN	2	1	0.1886	HGDN
15	HGDN	2	0	0.0281	HGDN
16	HGDN	2	1	0.1886	HGDN
13/16 (81.25%)					
**No**	**Histology**	**IHC score**		**Predict by**	
		**LIFR**	**CD34**	**Vaule**	**LIFR + CD34**
1	WD-sHCC	1	3	0.9804	WD-sHCC
2	WD-sHCC	0	3	0.9940	WD-sHCC
3	WD-sHCC	0	3	0.9940	WD-sHCC
4	WD-sHCC	0	3	0.9940	WD-sHCC
5	WD-sHCC	1	3	0.9804	WD-sHCC
6	WD-sHCC	1	3	0.9804	WD-sHCC
7	WD-sHCC	1	3	0.9804	WD-sHCC
8	WD-sHCC	0	3	0.9940	WD-sHCC
9	WD-sHCC	1	3	0.9804	WD-sHCC
10	WD-sHCC	0	2	0.9539	WD-sHCC
11	WD-sHCC	0	3	0.9940	WD-sHCC
12	WD-sHCC	0	3	0.9940	WD-sHCC
13	WD-sHCC	0	2	0.9539	WD-sHCC
14	WD-sHCC	2	3	0.9375	WD-sHCC

(*Continued*)

No	Histology	IHC score		Predict by	
		LIFR	CD34	Vaule	LIFR + CD34
15	WD-sHCC	0	3	0.9940	WD-sHCC
16	WD-sHCC	1	3	0.9804	WD-sHCC
17	WD-sHCC	1	3	0.9804	WD-sHCC
18	WD-sHCC	0	2	0.9539	WD-sHCC
19	WD-sHCC	0	3	0.9940	WD-sHCC
20	WD-sHCC	1	3	0.9804	WD-sHCC
21	WD-sHCC	0	3	0.9940	WD-sHCC
21/21 (100%)					

HGDN, high grade dysplastic nodule; WD-sHCC, well differentiated small hepatocellular carcinoma; IHC score, immunohistochemical score. Optimal cut-off value determined from ROC analysis was 0.3393 for LIFR + CD34 combination.
